# MiR-30b Is Involved in the Homocysteine-Induced Apoptosis in Human Coronary Artery Endothelial Cells by Regulating the Expression of Caspase 3

**DOI:** 10.3390/ijms160817682

**Published:** 2015-07-31

**Authors:** Feng Li, Qi Chen, Xiaowei Song, Lei Zhou, Jianliang Zhang

**Affiliations:** 1 Department of Cardiology, Changhai Hospital, Second Military Medical University, Shanghai 200433, China; E-Mails: martinlf@163.com (F.L.); Syfchenqi@163.com (Q.C.); saker3104@163.com (X.S.); 2 Department of Cadre Recuperation, Navy Medical Service Sanatorium, Hangzhou 310002, China; E-Mail: zhoulei_venus@163.com

**Keywords:** homocysteine, miR-30b, caspase-3, apoptosis, endothelial cells

## Abstract

Homocysteine (Hcy) is an independent risk factor for a variety of cardiovascular diseases, such as coronary heart disease, hypertension, stroke, *etc*. There is a close relationship between the vascular endothelial cell apoptosis and these diseases. Recent studies have shown homocysteine can induce apoptosis in endothelial cells, which may be an important mechanism for the development of theses cardiovascular diseases. Although there are several reports about how the Hcy induces apoptosis in endothelial cells, the exact mechanism is not fully understood. MicroRNAs are small, non-coding RNA. Previous studies have shown that there is a close relationship between several microRNAs and cell apoptosis. However, there are no studies about the role of microRNAs in Hcy-induced apoptosis in endothelial cells so far. In this study, we constructed the model of homocysteine-induced apoptosis in human coronary artery endothelial cells (HCAECs) and found miR-30b was significantly down-regulated by 1 mmol/L Hcy. In addition, overexpression of miR-30b can improve the Hcy-induced apoptosis in HCAECs by downregulating caspase-3 expression. Therefore, miR-30b may play an important role in Hcy-induced apoptosis in endothelial cells.

## 1. Introduction

Homocysteine (Hcy) is an intermediate metabolite in the metabolic pathway of cysteine and methionine. Previous studies have revealed that high levels of serum Hcy are independent risk factors of a variety of cardiovascular diseases, such as coronary heart disease, hypertension and stroke [1,2,3,4]. As we all know, vascular endothelial cells have an important role in normal physiological processes including the secretion of a variety of biologically active substances, including chemokines and cell adhesion factor, mediating immune inflammatory reaction, adjusting coagulation and fibrinolysis system, regulating vascular relaxation and contraction, promoting angiogenesis, *etc*. There is a close relationship between dysfunction of endothelial cells and cardiovascular diseases [5]. Hcy can induce apoptosis in endothelial cells [6,7,8], and the apoptosis induced by Hcy in endothelial cells is regarded as the one of the mechanisms leading to these cardiovascular diseases. Previous studies have found that Hcy induces endothelial cells apoptosis mainly through the death receptor pathway, the mitochondrial apoptotic pathway and the endoplasmic reticulum stress pathway, but the exact mechanism for how Hcy induces apoptosis in endothelial cells is not fully understood.

MicroRNAs are a class of small, non-coding RNA, which can affect the expression of target genes in the post-transcriptional level, change intracellular protein groups, and regulate apoptosis, proliferation, differentiation, and other physiological functions [9]. The recent accumulation of research results indicates that microRNAs play a significant role in regulating endothelial cell apoptosis. As we all know, Dicer is a key enzyme involved in the maturation of microRNAs. Previous study has found that overexpression of Dicer in human umbilical vein endothelial cells (HUVECs) markedly decreases apoptosis upon serum withdrawal [10]. In addition to Dicer, several individual microRNAs have been identified to have a key role in regulating endothelial cell apoptosis. Liu and colleagues have demonstrated that silencing of miR-155 leads to a reduction of apoptosis and reactive oxygen species production in human brain microvessel endothelial cells [11]. Qin *et al.* reported that downregulation of miR-221/222 in HUVECs by ox-LDL resulted in endothelial cell apoptosis, whereas overexpression of miR-221/222 in HUVECs markedly decreased apoptosis induced by ox-LDL via Ets-1/p21 pathway [12]. Furthermore, several other microRNAs are also shown to be important in regulating endothelial cell apoptosis, including miR-19a, miR-26a, miR-495, miR-US25-1, miR-223, let-7, miR-126, miR-21, miR-590-5p, miR-513a-5p, miR-23a, miR-365, *etc*. [13,14,15,16,17,18,19,20,21,22,23,24]. Overall, these studies indicate that microRNAs are crucial for the regulation of endothelial cell apoptosis.

Previous studies have shown that microRNAs are involved in Hcy-induced cardiovascular dysfunctions, such as cardiac remodeling and stroke [25,26]. However, there are no studies about the role of microRNAs in Hcy-induced apoptosis in endothelial cells so far. To further understand the molecular mechanism of Hcy-induced apoptosis in HCAECs, providing new drug targets and therapeutic orientation of intervention Hcy-induced endothelial dysfunction, the present study determined the role of miR-30b in Hcy induced apoptosis in the vascular endothelial cells.

## 2. Results

### 2.1. Hcy Induce Apoptosis in HCAECs by Upregulating the Expression of Caspase-3 in a Dose-Dependent Manner

**Figure 1:**
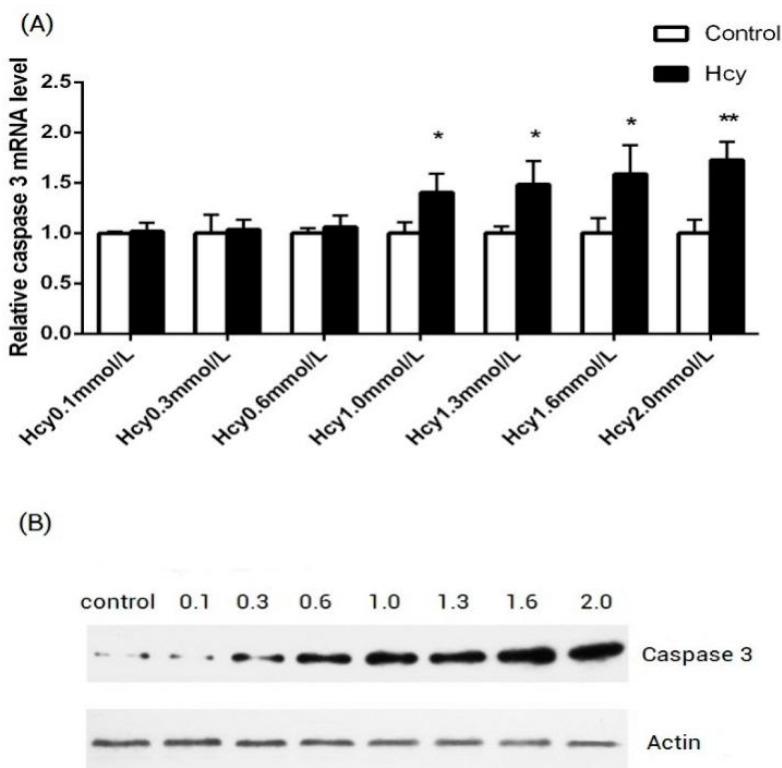
(**A**) The impact on expression of caspase 3 mRNA by different concentrations Hcy on HCAECs. The time of treatment was 24 h. Compared with the control group, * *p* < 0.05, ** *p* < 0.01, *n* = 3 in each group; and (**B**) The impact on expression of caspase 3 protein by different concentrations Hcy on HCAECs. The time of treatment was 24 h. From left to right is the control group, Hcy 0.1 mmol/L group, Hcy 0.3 mmol/L group, Hcy 0.6 mmol/L group, Hcy 1.0 mmol/L group, Hcy 1.3 mmol/L group, Hcy 1.6 mmol/L group, Hcy 2.0 mmol/L group.

**Figure 2:**
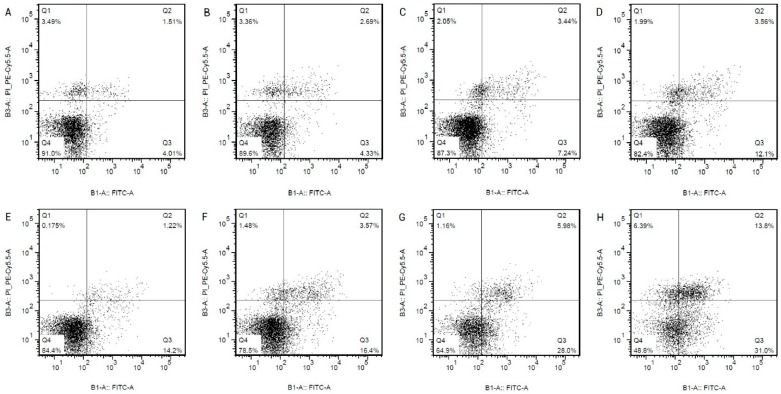
The apoptosis rate of different concentrations Hcy induced on HCAECs by flow cytometry in each group. The time of treatment was 24 h. (**A**–**H**), respectively, on behalf of the control group, Hcy 0.1 mmol/L group, Hcy 0.3 mmol/L group, Hcy 0.6 mmol/L group, Hcy 1.0 mmol/L group, Hcy 1.3 mmol/L group, Hcy 1.6 mmol/L group, Hcy 2.0 mmol/L group.

The HCAECs were treated with the Hcy of 0.1, 0.3, 0.6, 1.0, 1.3, 1.6 and 2.0 mmol/L concentration for 24 h. To observe the influence of Hcy on the HCAECs, the expression of caspase-3 mRNA was detected by real-time qPCR (Figure 1A), the expression of caspase-3 protein was detected by Western Blot (Figure 1B), the apoptosis rate was detected by flow cytometry with AnnexinV/PI staining (Figure 2 and Table 1). It was shown in Figure 1A that 1 mmol/L and above Hcy can significantly increase the expression of the caspase-3 mRNA (1.40-fold for 1 mmol/L Hcy, 1.48-fold for 1.3 mmol/L Hcy, 1.58-fold for 1.6 mmol/L Hcy, 1.72-fold for 2 mmol/L Hcy *vs.* control group, *p* < 0.05, respectively), while 0.1, 0.3 and 0.6 mmol/L Hcy did not significantly increase, compared with the control group. The Western Blot results were shown in Figure 1B that Hcy can increase the expression of the caspase-3 protein in a dose-dependent manner. To confirm the apoptosis of Hcy-induced in HCAECs, the apoptosis rate was detected and the results were shown in Figure 2 and Table 1. Hcy concentrations of 0.3 mmol/L and higher can significantly increase apoptosis in HCAECs (9.88% ± 0.86% for 0.3 mmol/L Hcy, 15.35% + 0.41% for 0.6 mmol/L Hcy, 16.28% ± 0.44% for 1 mmol/L Hcy, 19.72% ± 0.46% for 1.3 mmol/L Hcy, 32.9% ± 0.54% for 1.6 mmol/L, 48.76% ± 3.08% for 2 mmol/L Hcy *vs.* 5.92% ± 0.34% for control, *p* < 0.05).

**Table 1:** Apoptosis rate of different concentrations of Hcy-induced in HCAECs.

Group (*n* = 3)	Apoptosis Rate % (LR + UR)
control	5.92 ± 0.34
Hcy 0.1 mmol/L	6.5 ± 0.32
Hcy 0.3 mmol/L	9.88 ± 0.86 *
Hcy 0.6 mmol/L	15.35 + 0.41 **
Hcy 1.0 mmol/L	16.28 ± 0.44 **
Hcy 1.3 mmol/L	19.72 ± 0.46 **
Hcy 1.6 mmol/L	32.9 ± 0.54 **
Hcy 2.0 mmol/L	48.76 ± 3.08 **

Compare to the control group, * *p* < 0.05, ** *p* < 0.01 (*n* = 3 in each group); Abbreviations of the samples means: LR, Low right quadrants in the apoptosis figure of the flow cytometry, UR, Up right quadrants in the apoptosis figure of the flow cytometry.

### 2.2. MiR-30b Is Downregulated during Hcy-Induced Apoptosis in HCAECs

**Figure 3:**
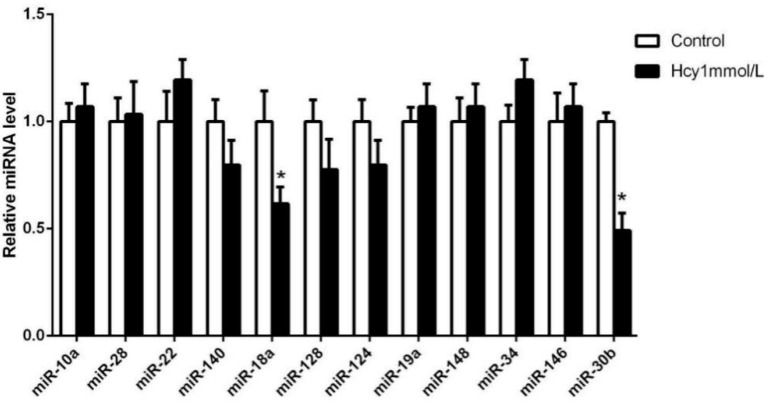
The expression of microRNAs in apoptosis Hcy-induced in HCAECs. The time of treatment was 24 h. Compared with the control group, * *p* < 0.05, *n* = 3 in each group.

In the first part of this study, we found that 1 mmol/L Hcy can significantly increase the expression of caspase-3 mRNA by real-time qPCR (Figure 1A). So, the HCAECs were treated with 1 mmol/L Hcy for 24 h and several microRNAs which expressed in endothelial cells were detected by real-time qPCR (Figure 3). MiR-30b is downregulated 0.49-fold during Hcy-induced apoptosis in HCAECs, compared with control group (*p* < 0.05).

### 2.3. Overexpression of miR-30b Inhibits the Apoptosis Induced by Hcy in HCAECs

**Figure 4:**
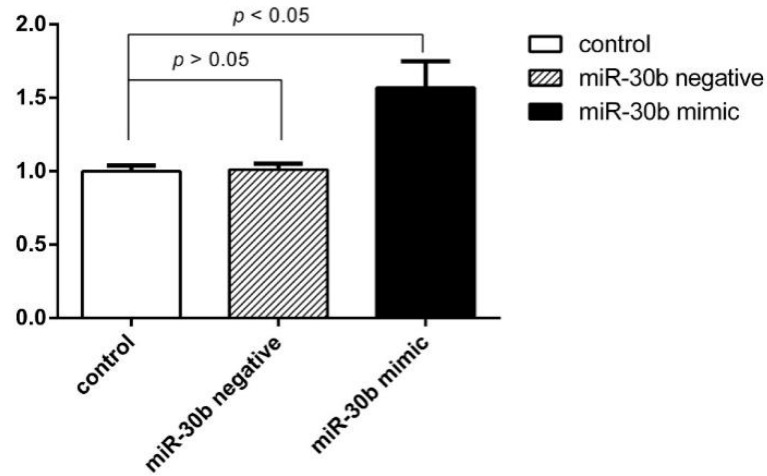
The expression of miR-30b upregulated after transfection of miR-30b mimic in HCAECs. The time of transfection was 24 h. Compared with the control group, the negative control group, *p* < 0.05 (*n* = 6 in each group).

**Figure 5:**
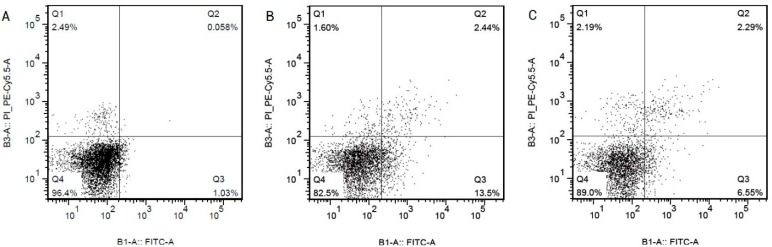
AnnexinV/PI flow cytometry apoptosis chart, on behalf of the control group (**A**); Hcy negative control group (**B**); and Hcy miR-30b overexpressed group (**C**). The concentration of Hcy was 1 mmol/L, both the time of transfection and treatment of Hcy were 24 h (*n* = 4 in each group).

**Figure 6:**
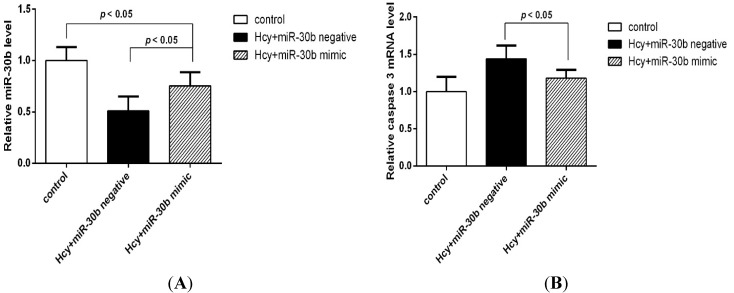
The expression of miR-30b and caspase-3 mRNA after overexpression of miR-30b in Hcy-induced HCAECs. The concentration of Hcy was 1 mmol/L, both the time of transfection and treatment of Hcy were 24 h. (**A**) shows the transfection of miR-30b mimic significantly up-regulated the expression of miR-30b which Hcy reduced; and (**B**) shows the overexpression of miR-30b down-regulated the expression of caspase-3 mRNA which Hcy induced. Compared with the control group, compared with the Hcy + miR-30b negative group, *p* < 0.05, *n* = 6 in each group.

**Figure 7:**
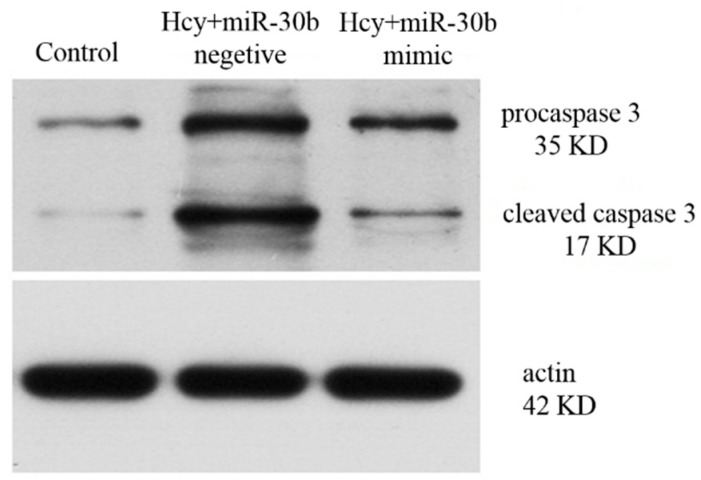
The expression of procaspase-3 and cleaved caspase-3 protein after overexpression of miR-30b in Hcy-induced HCAECs, the concentration of Hcy was 1 mmol/L, both the time of transfection and treatment of Hcy were 24 h.

To investigate the role of miR-30b in Hcy-induces apoptosis in the HCAECs, we upregulated the expression of miR-30b by transfecting with miR-30b mimics in HCAECs. When 100 ng miR-30b mimics were transfected into HCAECs, the level of miR-30b increased up to 1.5-fold compared with control group and miR-30b negative group, *p* < 0.05 (Figure 4). The HCAECs were treated with 1 mmol/L Hcy for 24 h after confirming the success and efficiency of the miR-30b transfection. It was shown in Figure 5 and Table 2 that overexpression of miR-30b in HCAECs significantly improved the Hcy-induced apoptosis (10.59% ± 0.6% for Hcy miR-30b overexpressed group, 15.83% ± 0.51% for Hcy negative group, 4.77% ± 1.23% for control group, *p* < 0.05). The influence of miR-30b overexpression on caspase-3 was also detected by real-time qPCR and Western blot. It was shown that the upregulation of caspase-3 by Hcy was significantly suppressed by miR-30b mimics both in mRNA level (Figure 6) and protein level (Figure 7). The increased expression of the cleaved caspase-3 induced by Hcy, which represented the activity of the caspase-3, was also significantly reduced by miR-30b mimics (Figure 7). The results of the Figure 7 are listed in Supplementary Tables S1 and S2. These data suggest that miR-30b plays an important role in the regulation of endothelial cell apoptosis induced by Hcy *in vitro*.

**Table 2:** Apoptosis rate of Hcy-induced in HCAECs.

Group (*n* = 4)	Apoptosis Rate % (LR + UR)
Control group	4.77 ± 1.23
Hcy negative control group	15.83 ± 0.51 *
Hcy miR-30b overexpressed group	10.59 ± 0.6 *^,#^

Compared with the control group, * *p* < 0.05; Compared with the negative control group, ^#^, *p* < 0.05 (*n* = 4 in each group); Abbreviations of the samples means: LR, Low right quadrants in the apoptosis figure of the flow cytometry; UR, Up right quadrants in the apoptosis figure of the flow cytometry.

## 3. Discussion

Since McCully found a close relationship between the levels of serum Hcy and atherosclerosis by autopsy in 1969 [27], the vascular complications by Hcy-induction raises concern. There is a close relationship between Hcy and cardiovascular diseases, and the endothelial cells’ apoptosis induced by Hcy is regarded as one of the most important mechanisms.

MicroRNAs are a class of small, non-coding RNA discovered in recent years. Accumulating evidence suggests important roles of microRNAs in the regulation of cell apoptosis. MiR-495 regulates proliferation and apoptosis in HUVECs by targeting the chemokine CCL2 [15]. Platelet-secreted microRNA-223 promotes endothelial cell apoptosis induced by advanced glycation end products via targeting the insulin-like growth factor 1 receptor [17]. Let-7g can inhibit oxidation of LDL-induced apoptosis in endothelial cell by regulating the expression of target gene caspase-3 [18]. However, there is no study reported about the role of microRNAs in Hcy-induced endothelial cell apoptosis so far.

To observe the role of microRNA in Hcy-induced apoptosis in endothelial cells, the Hcy-induced apoptosis model was constructed in HCAECs. For now, the majority of the previous apoptosis models were constructed on HUVECs, several were constructed on rat aortic endothelial cells and microvascular endothelial cells. Human vascular complications Hcy-induced mainly occurred in artery, so the HCAECs apoptosis model used in this study was more in accordance with the actual physiological situation of the body, compared with common HUVEC apoptosis models. Caspase-3 is a member of the cysteine-aspartic acid protease (caspase) family. It is the most important nucleic acid-cleaving enzyme of the apoptosis pathways, which plays an irreplaceable role in the cell apoptosis. So, the expression of caspase-3 was used to evaluate Hcy-induced apoptosis in HCAECs in this study. Previous studies have found that 500 µmol/L–1 mmol/L Hcy can significantly increase the apoptosis in HUVECs [7,8]. In our study, the expression of caspase-3 mRNA was significantly upregulated by 1 mmol/L Hcy, and the results were consistent with the previous date. Kerkeni M reported that the HUVECs can be treated with 10 mmol/L Hcy [28], but in our pre-experiment, almost all the HCAECs were not viable when treated with 3 mmol/L Hcy. These findings suggest that HCAECs have similar sensitivity but less tolerance for Hcy, compared with HUVECs.

In this study, several microRNAs which were in abundance in endothelial cells were detected by real-time qPCR. The miR-30b was significantly downregulated by 1 mmol/L Hcy. Previous studies have shown that miR-30b is involved in cell apoptosis by regulating the expression of caspase-3. Li constructed the ischemia-reperfusion (I/R) injury model in H2C9 rat myocardial cells and found the overexpression of miR-30b down-regulated the expression of caspase-3 and improve I/R injury in H2C9 cells [29]. Similarly, Song *et al.*, demonstrated that miR-30b levels were down-regulated by I/R injury in rat myocardial cells. Furthermore, overexpression of miR-30b inhibited myocardial cells apoptosis and exhibited a dramatic reduction of caspase-3 expression [30]. Zhu reported that miR-30b was significantly downregulated in human gastric cancer tissues, and overexpression of miR-30b in SGC-7901 or HGC-27 tumor cells can significantly promote cell apoptosis compared with the control group [31]. Liao found the expression level of miR-30b is low in colon cancer tissues, and the lower the level of miR-30b, the worse the differentiation and prognosis of colon cancer; overexpression of miR-30b can inhibit the proliferation and promote apoptosis of cancer cells apoptosis [32]. These studies indicate that miR-30b has an important role in regulating cell apoptosis and links with the caspase-3 expression. Because caspase-3 was the main indicator of apoptosis detected in this study, we chose miR-30b for further research. In addition to miR-30b, miR-18a was also significantly downregulated by 1 mmol/L Hcy in our study, and several researches had reported the important role of miR-18a in regulating cell apoptosis [33,34,35]. We believe that there should be more microRNAs involved in the process of the apoptosis Hcy-induced on HCAECs. Unfortunately, in this study, we only discussed the function and mechanism of miR-30b.

To further investigate the role of miR-30b in Hcy-induces apoptosis in HCAECs, miR-30b mimics were transfected into the HCAECs. Compared with the control group, the expression of miR-30b which was detected by real-time qPCR was significantly upregulated. Overexpression of miR-30b can inhibit the Hcy-induced apoptosis by downregulating the expression of caspase-3. Our study indicated that miR-30b was involved in the Hcy-induced apoptosis in HCAECs by regulating the expression of caspase-3. Interestingly, previous study has reported that Hcy-thiolactone which is a metabolite of Hcy, but not Hcy, induces apoptotic death in HUVECs in a caspase-3 independent way [36]. Furthermore, a recent article indicates that each of Hcy metabolites including Hcy-thiolactone and *N*-Hcy-protein induces a specific pattern of gene expression [37]. Are the changes of gene expression at high Hcy concentrations in our study are due to the accumulation of Hcy-thiolactone and/or *N*-Hcy-protein? Unfortunately, we did not carry out further research and analysis on this issue. Perhaps, in the future course of experiments, we will confirm this hypothesis.

## 4. Experimental Section

### 4.1. Reagents and Cell Cultures

HCAECs were kindly provided by Dr. Q.Jing (The Changhai Hospital, Shanghai, China) and cultured in ECM endothelial growth medium (ScienCell, San Diego, CA, USA) supplemented with 5% FBS and 100 U/mL penicillin/streptomycin at 37 °C under 5% CO2. d,l-Hcy (Sigma, Saint Louis, MO, USA) was dissolved in the ECM Medium. After the HCAECs grew to about 80% confluence, the cells were treated with Hcy of various concentration (0.1 mmol/L, 0.3 mmol/L, 0.6 mmol/L, 1.0 mmol/L, 1.3 mmol/L, 1.6 mmol/L, 2.0 mmol/L) for 24 h. Post Hcy incubation, the cells were collected for the apoptosis rate detection, mRNA expression analysis and Western blotting analysis.

### 4.2. Apoptosis Assays

The apoptosis rate was determined by flow cytometry with AnnexinV/PI staining (BD Biosciences, San Jose, CA, USA). Briefly, the HCAECs of each group were collected in 1.5 mL eppendorf, and washed by binding buffer. Each eppendorf contains about 1–5 × 10^5^ cells. The cells were resuspended in 100 µL and incubated with annexin V-FITC for 15 min in the dark at room temperature. Then, each eppendorf cells washed by binding buffer again and resuspended in 200 µL. After each group of cells were incubated with PI for 1 min, the apoptosis rates of Hcy-induced in HCAECs were detected by flow cytometry.

### 4.3. RNA Isolation, Reverse Transcription, Real-Time qPCR

Each group cells were lyzed by Trizol (Invitrogen, Carlsbad, CA, USA) and total cellular RNA were collected. RNA electrophoresis and densitometry were used to identify RNA quality. For quantitative analysis of caspase-3 mRNA expression and microRNAs expression, the special primers of caspases-3 and microRNAs (BioSune, Shanghai, China) were used in the follow-up experiments. Primer sequences are shown in the Table 3 and Table 4. The cellular RNA of each group (100–500 ng) was reversed into cDNA in reverse transcription reaction. The total reaction system is 10 µL and the reaction conditions were 16 °C for 30 min, 42 °C for 90 min, 72 °C for 5 min. Then, the reverse transcription products were amplified for real-time qPCR reaction after which were diluted 10 folds with PCR water. The conditions of real-time qPCR were 94 °C for 30 s, 60 °C for 30 s, 72 °C for 30 s, with 40 cycles in total. Relative quantification was determined using the ΔΔ*C*_t_ method, U6 was used as reference gene.

**Table 3:** Primer for reverse transcription.

Primer Name	Primer Sequence
hsa-miR-10a-5p RT primer	5′-GTCGTATCCAGTGCAGGGTCCGAGGTATTCGCACTGGATACGACCACAAATT-3′
hsa-miR-28-5P RT primer	5′-GTCGTATCCAGTGCAGGGTCCGAGGTATTCGCACTGGATACGACCTCAAT-3′
hsa-miR-22-5p RT primer	5′-GTCGTATCCAGTGCAGGGTCCGAGGTATTCGCACTGGATACGACTAAAGC-3′
hsa-miR-140-5p RT primer	5′-GTCGTATCCAGTGCAGGGTCCGAGGTATTCGCACTGGATACGACCTACCA-3′
hsa-miR-18a-5p RT primer	5′-GTCGTATCCAGTGCAGGGTCCGAGGTATTCGCACTGGATACGACCTATCT-3′
hsa-miR-128 RT primer	5′-GTCGTATCCAGTGCAGGGTCCGAGGTATTCGCACTGGATACGACAAAGAG-3′
hsa-miR-124a RT primer	5′-GTCGTATCCAGTGCAGGGTCCGAGGTATTCGCACTGGATAC-3′
hsa-miR-19a RT primer	5′-GTCGTATCCAGTGCAGGGTCCGAGGTATTCGCACTGGATACGACTCAGTTTT-3′
hsa-miR-148b RT primer	5′-GTCGTATCCAGTGCAGGGTCCGAGGTATTCGCACTGGATACGACACAAAGTT-3′
hsa-miR-34b RT primer	5′-GTCGTATCCAGTGCAGGGTCCGAGGTATTCGCACTGGATACGACCAATCA-3′
hsa-miR-146a RT primer	5′-GTCGTATCCAGTGCAGGGTCCGAGGTATTCGCACTGGATACGACAACCCA-3′
hsa-miR-30b-5p RT primer	5′-GTCGTATCCAGTGCAGGGTCCGAGGTATTCGCACTGGATACGACAGCTGA-3′

**Table 4:** Primer for real-time qPCR.

Primer Name	Primer Sequence
hsa-miR-10a-5p Forward primer	5′-ACGGGTACCCTGTAGATCCG-3′
hsa-miR-28-5P Forward primer	5′-GCCAAAGGAGCTCACAGTCT-3′
hsa-miR-22-5p Forward primer	5′-AGGCAGTTCTTCAGTGGCAA-3′
hsa-miR-140-5p Forward primer	5′-ACGGGCAGTGGTTTTACCCTA-3′
hsa-miR-18a-5p Forward primer	5′-AGGGCTAAGGTGCATCTAGTGC-3′
hsa-miR-128 Forward primer	5′-ATCCTTCACAGTGAACCGGT-3′
hsa-miR-124 Forward primer	5′-GTGAATTAAGGCACGCGGTG-3′
hsa-miR-19a Forward primer	5′-ACGCCTGTGCAAATCTATGC-3′
hsa-miR-148b Forward primer	5′-CGGCTCAGTGCATCACAG-3′
hsa-miR-34b Forward primer	5′-ACGGGTAGGCAGTGTCATTAGC-3′
hsa-miR-146a Forward primer	5′-AGCCGTGAGAACTGAATTCCA-3′
hsa-miR-30b-5p Forward primer	5′-ACGGGCTGTAAACATCCTACAC-3′
has-miR Reverse primer	5′-CAGTGCAGGGTCCGAGGTAT-4′
caspase 3 Forward primer	5′-TGTGAGGCGGTTGTGGAAGAGT-3′
caspase 3 Reverse primer	5′-AATGGGGGAAGAGGCAGGTGCA-3′
U6 Forward primer	5′-CTCGCTTCGGCAGCACA-3′
U6 Reverse primer	5′-AACGCTTCACGAATTTGCGT-3′

### 4.4. Western Blot

Each group cells were lyzed with Radio Immunoprecipitation Assay (RIPA) cell lysis reagent (Beyotime, Shanghai, China) and the cell protein was collected in 1.5 mL eppendorf. The protein concentration was detected by Bradford Reagent (Amresco, Solon, OH, USA). After the preparation of the SDS-PAGE separating gel and stacking gel, the target protein samples were added 40 µg to per well, transferred to a polyvinylidene fluoride (PVDF) membrane under conditions 200 mA for 1 h and blocked in 5% skimmed milk. Then, the PVDF membrane was incubated overnight at 4 °C with anti-β-actin (Sigma, Saint Louis, MO, USA), anti-caspase-3 antibody (Cell Signaling Technology, Shanghai, China) diluted by Tris-Buffered Saline and Tween (TBST). After washing membrane, incubating with secondary antibody (Cell Signaling Technology, Shanghai, China), washing membrane again, the sample protein quantification was analyzed in odyssey equipment. The β actin was used as reference genes.

### 4.5. Overexpression of miR-30b

MiR-30b microRNA mimics (GenePharma, Shanghai, China) were utilized to overexpress the miR-30b. In order to analysis of miR-30b suppressing apoptosis, 100 ng miR-30b mimics or miR-30b negative control was transfected into HCAECs with FuGENE HD transfection reagent (Roche, Basel, Switzerland) for 24 h. The success and efficiency of transfection was confirmed by real-time qPCR. After the transfection, the cells were treated with 1 mmol/L Hcy for another 24 h, then the expression of caspase-3 and apoptosis rate were analyzed.

### 4.6. Statistical Methods

Data were expressed as mean ± standard deviation, *t*-test was used to compare the experimental group and the control group, one-way ANOVA was used to compare the control group, miR-30b negative group/Hcy + miR-30b negative group, miR-30b mimic group/Hcy + miR-30b mimic group. *p* < 0.05 was considered significant differences.

## 5. Conclusions

We found that Hcy can induce apoptosis in HCAECs in a dose-dependent manner. In this process, the expression of caspase-3 was upregulated and miR-30b was downregulated. In addition, overexpression of miR-30b inhibited apoptosis Hcy-induced in HCAECs by downregulating the caspase-3 expression. Therefore, miR-30b may play an important role in apoptosis induced by Hcy in endothelial cells.

## Supplementary Materials

Supplementary materials can be found at http://www.mdpi.com/1422-0067/16/08/17682/s1.
