# Supplementary material for: MiR-30b Is Involved in the Homocysteine-Induced Apoptosis in Human Coronary Artery Endothelial Cells by Regulating the Expression of Caspase 3
Source: Int J Mol Sci. 2015 Jul 31;16(8):17682–95. doi: 10.3390/ijms160817682 (PMC4581215; doi:10.3390/ijms160817682)
Supplement: Supplementary file 1 [file ijms-16-17682-s001.pdf]

Supplementary Information

Table S1. The Gray value of the sample in Figure 7.

Groups	β Actin	Cleaved Caspase 3
Control	365,424	63,000
Hcy + miR-30b negative	352,720	242,172
Hcy + miR-30b mimic	357,000	136,416

Table S2. The gray value ratio of the β actin and clever caspase 3.

Groups	Ratio (Clever Caspase 3/β Actin)
Control	0.17
Hcy + miR-30b negative	0.69
Hcy + miR-30b mimic	0.38
